# A Multiple-Hit Hypothesis Involving Reactive Oxygen Species and Myeloperoxidase Explains Clinical Deterioration and Fatality in COVID-19

**DOI:** 10.7150/ijbs.51811

**Published:** 2021-01-01

**Authors:** Pravin T Goud, David Bai, Husam M Abu-Soud

**Affiliations:** 1 Division of Reproductive Endocrinology and Infertility & California IVF Fertility Center, Department of Obstetrics and Gynecology, University of California Davis, Sacramento, CA, 95833, USA.; 2 California Northstate University Medical College, Elk Grove, CA, 95757, USA.; 3 Department of Obstetrics and Gynecology, The C.S. Mott Center for Human Growth and Development, Wayne State University School of Medicine, Detroit, MI, 48201, USA.; 4 Department of Physiology, Wayne State University School of Medicine, Detroit, MI, 48201, USA.; 5 Department of Microbiology, Immunology and Biochemistry, Wayne State University School of Medicine, Detroit, MI, 48201, USA.

**Keywords:** Coronavirus. COVID-19, reactive oxygen species, free iron, myeloperoxidase, HOCl

## Abstract

Multi-system involvement and rapid clinical deterioration are hallmarks of coronavirus disease 2019 (COVID-19) related mortality. The unique clinical phenomena in severe COVID-19 can be perplexing, and they include disproportionately severe hypoxemia relative to lung alveolar-parenchymal pathology and rapid clinical deterioration, with poor response to O_2_ supplementation, despite preserved lung mechanics. Factors such as microvascular injury, thromboembolism, pulmonary hypertension, and alteration in hemoglobin structure and function could play important roles. Overwhelming immune response associated with “cytokine storms” could activate reactive oxygen species (ROS), which may result in consumption of nitric oxide (NO), a critical vasodilation regulator. In other inflammatory infections, activated neutrophils are known to release myeloperoxidase (MPO) in a natural immune response, which contributes to production of hypochlorous acid (HOCl). However, during overwhelming inflammation, HOCl competes with O_2_ at heme binding sites, decreasing O_2_ saturation. Moreover, HOCl contributes to several oxidative reactions, including hemoglobin-heme iron oxidation, heme destruction, and subsequent release of free iron, which mediates toxic tissue injury through additional generation of ROS and NO consumption. Connecting these reactions in a multi-hit model can explain generalized tissue damage, vasoconstriction, severe hypoxia, and precipitous clinical deterioration in critically ill COVID-19 patients. Understanding these mechanisms is critical to develop therapeutic strategies to combat COVID-19.

## Introduction

Coronavirus disease 2019 (COVID-19), a disease caused by severe acute respiratory syndrome coronavirus 2 (SARS-CoV-2), has shaken the world with its rapid and continual global spread in pandemic proportions 1. Moreover, the economic and social impact of the pandemic is enormous and unprecedented. Currently, over 33 million individuals around the world have been affected, and over 1 million have succumbed to the severe respiratory distress associated with COVID-19 or related complications 2. In particular, the United States continues to lead the world in cases, with over 7 million infected and at least 200,000 dead as of the time of writing 3. Clinical management of COVID-19 is uncertain, especially due to the novelty of the virus and poorly understood pathophysiology, combined with the urgency of the situation limiting time available for clinical trials and further testing 4-9. As a result, as consensus guidelines for COVID-19 treatment emerge, many clinicians are presently focusing on supportive treatments such as ventilator support 10. However, mortality among the hospitalized cases continues to be high. Administration of oxygen and support via mechanical ventilation sometimes fail to prevent the profound hypoxia associated with COVID-19 from causing significant clinical decline 10, 11. Thus, the pathophysiology of COVID-19 may be unique compared to known respiratory conditions such as typical acute respiratory infections, pneumonia, or acute respiratory distress syndrome (ARDS) 11, 12.

Published reports indicate that approximately 20% of COVID-19 patients tend to have severe or critical disease, with a mortality rate of 50% or more in critical cases 13-17. According to various reports, COVID-19 mortality has been predicted by factors such as decreased hemoglobin, elevated cytokines, D-dimer, cardiac and/or renal injury, leukocytosis, and elevation of neutrophil : lymphocyte ratio (NLR) 16, 17. One possible explanatory aspect for these factors contributing to severe COVID-19 cases is an inappropriately exaggerated immune response previously described as a “cytokine storm,” which results in the formation of reactive oxygen species (ROS) 18-20. Such an acute severe immunological response in similar viral infections is known to release a cascade of inflammatory mediators, including various interleukins, tumor necrosis factor (TNF α) and other chemokines. Interleukin-6 (IL-6), which is capable of signaling through both membrane-bound receptors (IL-6R) and soluble receptors, is one of the most important 21. The IL-6 pathway involves increased vascular permeability and immune cell recruitment through modulation of endothelial activation and dysfunction 21. Furthermore, infiltrating neutrophils, a hallmark of COVID-19, can release myeloperoxidase (MPO), which can activate several pathways that lead to elevated cytokines and production of ROS such as hypochlorous acid (HOCl), superoxide (O_2_•^-^), and hydrogen peroxide (H_2_O_2_) 22-24. Notably, HOCl can both compete with O_2_ at hemoglobin heme binding sites and also cause heme degradation and subsequent release of free iron (Fe^2+^). Free iron can then undergo the Fenton reaction to produce an array of ROS, including the highly reactive hydroxyl radical (•OH) 23-27. Another possible facet of the observed pathophysiology in critical cases of COVID-19 is a decline in nitric oxide (NO), a key mediator of vasodilation 28, 29. Vasodilation mediated by NO deficiency combined with the effect of excessive ROS on the structure and function of hemoglobin (Hb) could impact pulmonary and peripheral circulation, possibly eventually leading to critical or fatal hypoxia. Therefore, connection of these pathways establishes a multi-hit model that relates simultaneous activation of ROS-mediated mechanisms to key features of critically ill COVID-19 patients. Understanding these pathways will provide multiple clues to develop therapeutic strategies that will prevent severe morbidity and mortality related to COVID 19.

## Pathophysiology of COVID-19

SARS-CoV-2 is a positive sense singled-stranded RNA virus with a nucleocapsid 30. Human-to-human transmission occurs via respiratory droplets imparted by close contact, contaminated surfaces, or aerosol in closed spaces 22. Animal-to-human transmission has been speculated on, but the evidence remains insubstantial. SARS-CoV-2 is thought to enter the host cells via angiotensin converting enzyme-2 (ACE-2) receptors ubiquitously expressed on the cells of the lungs, gastrointestinal tract, blood vessels, heart, and kidney 31. The primary target of SARS CoV-2 appears to be the lower respiratory tract, rather than the ciliated epithelial cells of the conducting airways; cells in the lower respiratory tract are indeed known to express ACE-2 receptors 32. Once inside host cells, the virus hijacks cellular machinery for viral replication, ultimately leading to cellular destruction and further proliferation of virions 32, 33.

Current speculations on the immediate host immune response to SARS-CoV-2 is based mostly on previous research on SARS-CoV, Middle East respiratory syndrome related coronavirus (MERS-CoV), and other related viral infections. SARS-CoV is the most similar virus, showing 79% and 69% similarity in terms of genome and amino acid sequence, respectively, to SARS-CoV-2 34. Direct infection of macrophages and T cells may also be possible 32. As postulated based on SARS-CoV, the initial innate immunological response subsequent to viral entry into monocytes/macrophages begins with pathogen associated molecular patterns (PAMP) being recognized by the endosomal RNA receptors TLE3 and TLR7 and cytosolic RNA sensors RIG-1/MDA5, activating a downstream signaling cascade involving nuclear translocation of NF-κB and TRF3 causing transcription of type I interferon (IFN). This in turn activates IFNAR, phosphorylating STAT1 and STAT2 via the JAK-STAT pathway and has the final result of viral destruction through IFN 35. Based on information gathered from studies on SARS-CoV and MERS-CoV, a putative delayed or dysfunctional IFN-1 host response may result in neutrophil and monocyte-macrophage hyper response, especially in lethal cases of SARS CoV and MERS. Continued viral propagation can accordingly continually activate neutrophil and therefore cause release of proinflammatory cytokines in the aforementioned cytokine storm 20, 36. Other immunological responses include an adaptive immune response, where the cytokine milieu created by antigen presenting cells directs T cell responses in the form of helper T cell actions that orchestrate the overall adaptive immune response and cytotoxic T cell actions kill virus infected cells. Additionally, the humoral immune response results in seroconversion, with secretion of anti-IgM antibody as early as 4-14 days after onset of symptoms in patients with SARS-CoV 2 37, 38. The clinical course of COVID-19 begins with an asymptomatic or mildly symptomatic phase, when the disease could be locally progressive, and there may be some innate immune response. Currently, it is estimated that about 80% of patients suffer nothing more than mild to moderate upper respiratory symptoms 39. Meanwhile, progressive viral replication continues in individuals unable to clear the virus; these 20% of patients progress to the severe stage of COVID-19. This stage is marked by lower respiratory tract and lung symptoms, particularly infection of type II alveolar pneumocytes in the peripheral and subpleural lungs 40. Poor prognosis is generally marked by relative lymphopenia, elevated inflammatory markers characteristic of the cytotoxic storm, and markers of cardiac, renal or coagulation system anomaly 41. Tissue destruction through viral destruction and induced apoptosis then results in alveolar damage. Recovery is dependent on the specificity and degree of both the innate and adaptive immune response against the virus. However, the repair process itself could be aberrant, especially in case of a hyperimmune response and may result in fibrosis and lung damage, especially that associated with excessive ROS. Proper activation of cellular antioxidant machinery is important to regulate the high degree of stimulation of ROS pathways in the immune or hyperimmune response and prevent cellular damage.

## Generation of ROS at sites of inflammation

Typically, in COVID-19 and similar viral infections, the inflammatory immune response begins with chemokines released from inflamed respiratory tissue, followed by infiltration of leukocytes, especially neutrophils and macrophages 42, 43. In the earliest stages of the immune response, neutrophils in particular play a crucial role by neutralizing and destroying viral proteins with the aid of ROS such as O_2_•^-^, H_2_O_2_, •OH and HOCl, essential components of the immune response 44. In addition to their direct roles in the immune response, ROS can also act in cellular messaging by modifying the activity of immune and other cells; however, they can exert significant cytotoxic effects when released in the large amounts typical in some immune responses 45, 46. Consequently, understanding the activity of ROS in infection such as COVID-19 is critical to grasping the pathology.

**Figure 1:**
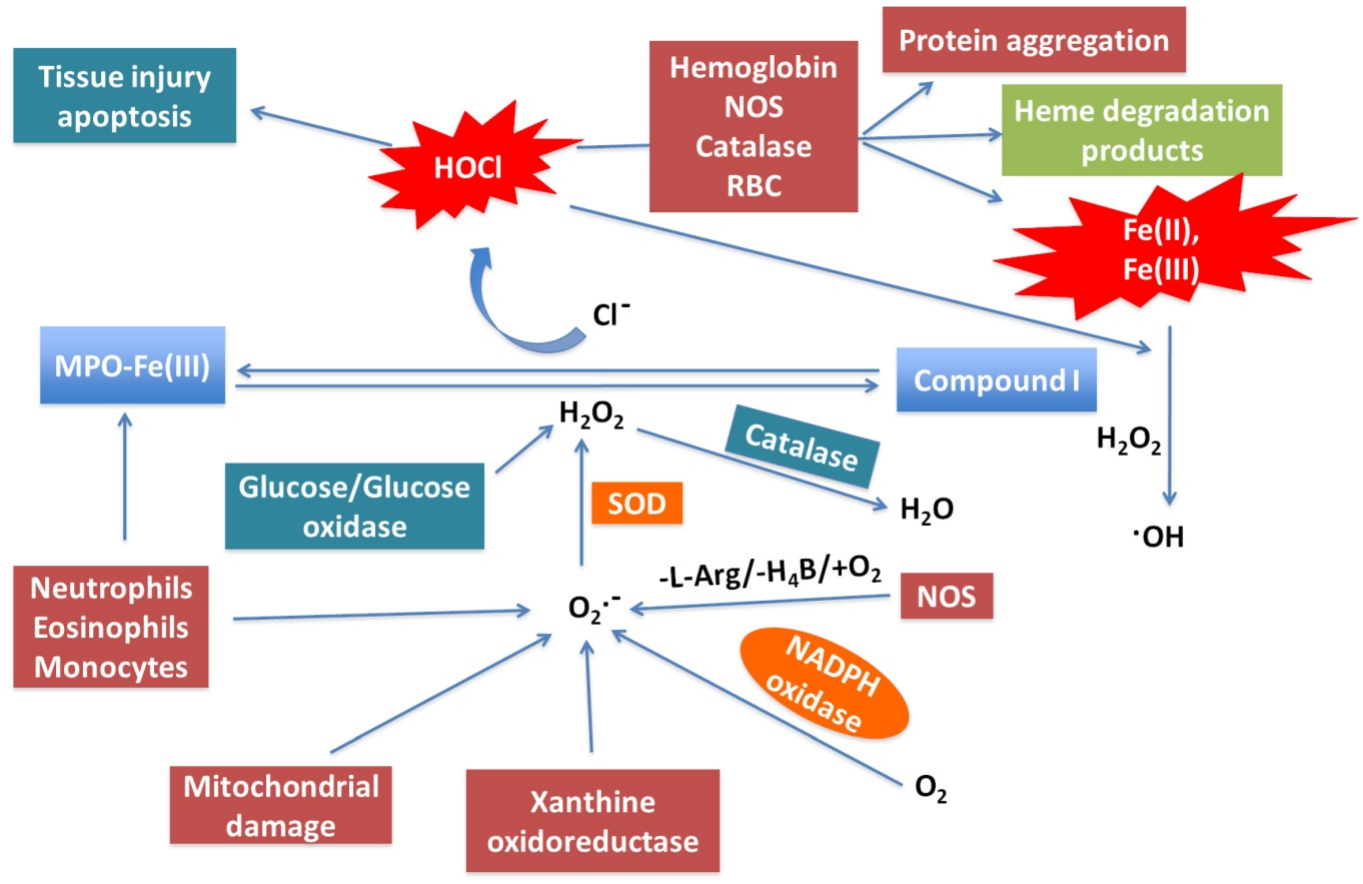
** Neutrophil MPO -generated oxidants and their potential role in COVID-19.** Activated neutrophils, eosinophils, and mitochondrial damage competently generate reduced O_2_ species such O_2_•^-^ and H_2_O_2_ by a process known as oxidative burst. MPO is secreted from activated neutrophil which is abundant in human blood. MPO utilizes H_2_O_2_ as an oxidative substrate. In the presence of plasma levels of chloride, the enzyme generates the oxidant HOCl. MPO, under normal conditions, releases ROS to eliminate invading microorganisms. Under abnormal conditions, the generated ROS is excessive and mediates DNA oxidative damage, tissue injury and apoptosis. It also mediates hemoprotein (e.g. hemoglobin, NOS, and catalase) heme destruction and release of heme degradation products, protein aggregation, and free iron release. Free iron (Fe^2+^/Fe^3+^) reacts through the Fenton reaction to generate the highly reactive and toxic hydroxyl radical. By generating ROS, consuming NO, mediating hemoprotein heme destruction, promoting free iron release and •OH generation, neutrophil MPO activity may contribute to COVID-19 pathology by promoting oxidative stress, vasoconstriction by consuming NO, hypoxia through hemoglobin heme destruction, and blood clotting by generating free iron, and DNA damage through generation of hydroxyl radical.

A model showing the link between neutrophil MPO activity generated during the “cytokine storm” provoked by COVID-19, ROS, and its role in NO consumption and heme destruction as well as subsequent iron release is shown in Figure 1. In this model, neutrophils, eosinophils, monocytes, macrophages, mitochondrial damage, and NADPH oxidase are the major sources of generation of O_2_^•-^ at sites of inflammation 47-50. Another ROS-generating enzyme is xanthine oxidoreductase (XOR), which metabolites hypoxanthine and xanthine to uric acid to instantaneously generate O_2_•^-^ 51. In COVID-19, like other inflammatory diseases, a major damaging pathway mediated by overproduction of O_2_•^-^ and subsequent oxidative stress is caspase-3 activation, which is closely associated apoptosis and therefore DNA fragmentation 52. However, O_2_•^-^ is a short-lived molecule and is quickly consumed either through nonenzymatic pathways or a superoxide dismutase-catalyzed reaction to produce H_2_O_2_ 53, 54. Meanwhile, H_2_O_2_ is considerably more stable than O_2_•^-^, diffuses freely through biological membranes, and is equally capable of inducing cytotoxicity when overproduced, as in COVID-19 55, 56. In addition to the dismutation of O_2_•^-^, enhancement of H_2_O_2_ during infection is also related to the activity of a variety of oxidases such as glucose/glucose, monoamine, and amino acid oxidase 57. In addition to its direct effects, H_2_O_2_ can also react with O_2_•^-^ or Fe^2+^, through the Fenton reaction, to generate the highly reactive and toxic hydroxyl radical. These can contribute to tissue damage, further worsening the condition of infected individuals 58. Catalase, a key regulator of H_2_O_2_, scavenges H_2_O_2_ and catalyzes its decomposition, thereby protecting cells from H_2_O_2_ toxicity 59.

## Myeloperoxidases could play a central role in COVID-19

**Figure 2:**
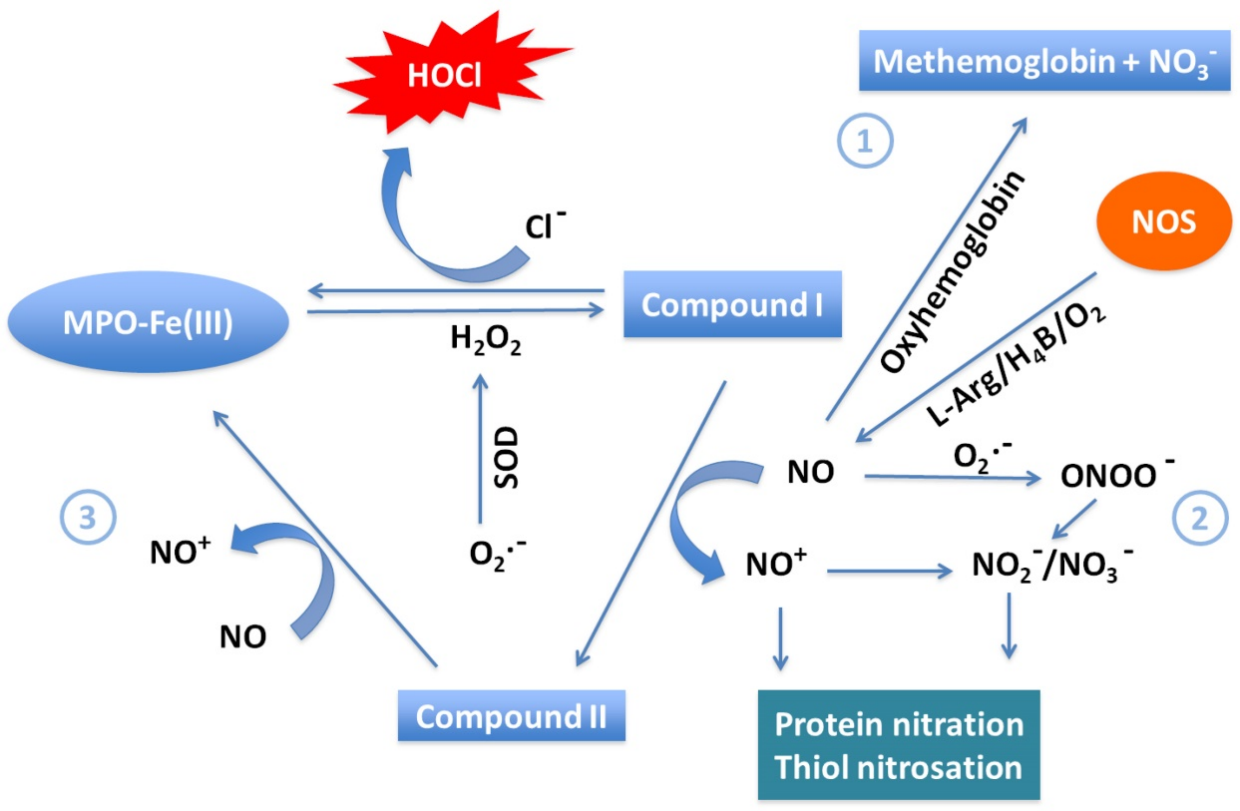
** Major pathways of NO scavenging under inflammatory conditions.** Three major pathways have been proposed to participate in consumption of NO in COVID-19. (1) The reaction of NO with oxy-hemoglobin to yield methemoglobin and NO_3_^-^ is likely a major pathway of NO consumption in vivo. (2) The near diffusion-controlled rate reaction of NO with O_2_•^-^ to yield ONOO^-^, which then decays to NO_3_^-^. This reaction is of specific importance wherever enhanced rates of both NO and O_2_•^-^ production take place. (3) Overproduction of MPO in COVID-19 efficiently consumes NO as a 1e^-^ substrate for both MPO Compounds I and II during steady-state catalysis, forming the very reactive NO^+^, which rapidly decays to NO_2_^-^. This pathway is particularly relevant at sites of inflammation where leukocyte peroxidases, NO, and H_2_O_2_ are present.

Secondary to initial viral entry of SARS-CoV-2 into alveolar cells, a substantial number of neutrophils infiltrate 60. They then release MPO, a major scavenger of H_2_O_2_ in the respiratory system, from azurophil granules. In the presence of Cl^-^, MPO catalyzes generation of HOCl from H_2_O_2_, contributing this important antimicrobial oxidant to the immune response 61, 62. However, HOCl is also a long-lived oxidative species that can be the source of hydroxyl radical, e.g. upon reaction with O_2_•^-^, and there is evidence documenting its destructive interactions with hemoproteins 63, 64. Moreover, MPO can utilize NO as a 1e- substrate for MPO Compound I and Compound II, generating nitrosonium cation (NO^+^) and nitrite (NO_2_^-^) as final products 65) (Figure 2). Consumption of NO, a vasodilator, through this pathway contributes to vasoconstriction of the pulmonary vasculature. Therefore, the MPO-HOCl system and other ROS associated with MPO may take part in accelerating lung damage in infected patients. Accumulation of these species in the blood of COVID-19 patients can cause destruction of hemoproteins and generate free iron, which further promotes oxidative stress, also hindering normal physiological function, while damaging vital lipids, proteins, and nucleic acids 25, 64, 66. A particularly significant hemoprotein affected by HOCl proliferation is Hb, which may be related to the hypoxia observed in COVID-19.

## Hypochlorous acid alters hemoglobin function

Recently, it has been reported that many COVID-19 patients had low peripheral O_2_ saturation (50 -70%) but absence of common symptoms of O_2_ deficiency such as shortness of breath, troubled breathing, or dizziness 67, 68. In COVID-19, a primary site of damage is the alveolar membrane, the site of O_2_ transfer from inspired air to blood. This is a possible explanation of the decreased O_2_ saturation in blood. However, when lung mechanics are preserved or the decrease of O_2_ saturation is disproportionate to the extent of lung damage, alternate possibilities must be considered. Here, neutrophil MPO activity may also contribute to O_2_ deprivation in these patients, rationalizing the phenomenon of patients with relatively low oxygen saturation without corresponding symptoms 67, 68.

Since CO_2_ levels are also low in COVID-19 patients, a defect in hemoglobin, the O_2_/CO_2_ carrier, is also possible 22. Hemoglobin is a tetramer of four heme groups attached to four globin subunits 69. These heme prosthetic groups transport O_2_ from the lung to peripheral tissue for cellular metabolism while ferrying CO_2_ back from peripheral tissue to the lung. To fulfill its function, Hb works against the concentration gradient by binding O_2_ in the oxygen-rich lungs to transport and release it in peripheral tissue, which are relatively O_2_-deficient 69. Human Hb exists mainly in two reduced forms: oxygenated (Hb-Fe(II)-O_2_, oxy-Hb) and deoxygenated (Hb-Fe(II)-CO_2_). HOCl can disrupt Hb function by oxidizing oxyHb to methemoglobin and binding to the ferric heme iron to form HB-Fe(III)-OCl complex that can then decay to Hb-Fe(IV)=O (Compound II) through the formation of Hb-Fe(IV)=O^+Π●^ (Compound I) and eventually lead to Hb heme destruction 64. Heme destruction releases a variety of degradation products, particular the toxic free iron, and also encourages aggregation of globins 64. Furthermore, methemoglobin and Hb ferryl intermediates are forms of Hb that cannot bind O_2_ 70. Therefore, accumulation of these species in blood during COVID-19 could result in tissue hypoxia through the lack of normal Hb function. Furthermore, it is notable that the affinity of Hb toward OCl^-^ is higher than the affinity of Hb toward O_2_, which further inhibits normal Hb function 64. Therefore, during early stages of the disease, O_2_ supplementation could be beneficial in reoxygenation of Hb and correcting O_2_ saturation. However, at later stages of disease, there would be accordingly higher OCl^-^ generation, which results in widespread, irreversible heme destruction of Hb. In this situation, supplementation with O_2_ could conversely be deadly due to additional generation of toxic ROS such as O_2_^•-^and H_2_O_2_.

HOCl-mediated cleavage reaction of the Hb heme and oxidation occurs randomly at any of the heme double bonds, independent of HOCl concentration. The degree of heme degradation depends mainly on the ratio of HOCl to heme, suggesting that multiple molecules of HOCl are required per molecule of heme. Additionally, excess ROS, especially O_2_^•-^ and H_2_O_2_, have also been reported to destroy the heme of Hb and other hemoproteins such as cytochrome c, catalase and NOS with a similar mechanism 71. Thus, inhibiting the chlorinating activity of MPO and/or direct scavenging of HOCl in the early stages of infection could be helpful to the patients with COVID-19. Recently, it has been shown that melatonin, lycopene, taurine, glutathione, and methionine not only serve as potent HOCl scavengers but also inhibit the chlorinating activity of MPO, thereby preventing HOCl-mediated heme destruction and subsequent free iron release 72, 73.

It is important to note that the mechanism of HOCl-mediated heme destruction completely differs from controlled heme oxygenase activity, which catalyzes the rate-limiting enzymatic step of heme degradation into carbon monoxide, free iron, and biliverdin 74. Heme oxygenase-1 (HO-1) is an antioxidant enzyme, and its expression and activity are significantly up-regulated at sites of viral inflammation. Deficiencies in HO-1 expression have been reported to modulate chronic inflammation in both mice and humans 75.

## Toxicity of free iron

The toxicity of extraneously generated free iron deserves special mention due to its ability to mediate the production of a variety of toxic ROS, such as the O_2_•^-^, H_2_O_2_, and •OH, that then cause cellular mitochondria poisoning, lipid peroxidation, damage to oxidative phosphorylation pathways 76, 77. Furthermore, free iron can both damage blood vessels and promote cardiovascular complications such as hypertension and metabolic acidosis through vasodilation and increased vascular permeability 78-80. Another cardiovascular condition free iron is associated with is increased coagulation through enhancement of fibrinogen activity, which is especially dangerous given the relationship between this phenomenon and thrombotic disease, i.e. stroke 81, 82. Finally, increased free iron accumulation has been reported in a variety of other pathologies such as atherosclerosis, endometriosis, and cancer, which have also been correlated with increased MPO activity 78, 83, 84. The enhanced MPO associated with these conditions further predisposes affected individuals towards both severe complications of COVID-19, other pathological conditions, and biochemical imbalances such as depletion of critical species, e.g., NO.

## MPO and other members of the mammalian peroxidase superfamily serve as catalytic sinks for NO at sites of infection

As NO is produced in lungs, it may diffuse into the lumen of blood vessels, where it will mostly be scavenged by Hb at near diffusion rates in a controlled interaction with erythrocyte oxyhemoglobin, yielding ferric (met)hemoglobin and nitrate (NO_3_-) 85. However, during advanced stages of COVID-19, hemoglobin levels and saturation are significantly decremented, and NO scarcity, vasoconstriction, and hypertension are present 86. In particular, these observations imply either decreased production or increased consumption of NO in the subendothelial space of lung tissue and other inflamed tissues 1. The reason for this has not yet been explicated, but a possible explanation is hypoxic suppression of NOS activity, in which low O_2_ ventilation (O_2_ concentration close to or lower than the K_m(O2)_ values of NOSs) depresses cellular O_2_ below the level required for NOS activity. Importantly, it has been shown that in some cases of COVID-19, Hb O_2_ can reach as low as 50%, which is indeed close to or lower than the Km_(O2)_ of both NOS-II and NOS-I 87. Under these conditions, NOS generates O_2_^•-^ instead of NO, which causes a cascade of other harmful effects.

Meanwhile, long term changes in NO levels through action of NO scavengers has been previously described. One major pathway for removal of NO in tissues is through its rapid reaction with O_2_•, producing peroxynitrite (ONOO^-^) 87. This reaction may be of particular importance at sites of inflammation and phagocyte activation where both NO and O_2_•^-^ production are elevated. However, NO removal only through this pathway does not explain the complete loss of NO-dependent signaling in vascular smooth muscle cells, suggesting alternative pathways of NO depletion 88.

Alternatively, the MPO-H_2_O_2_ system may also influence NO levels at sites of inflammation through two distinct mechanisms, namely, (1) consuming NO as a one e^-^ substrate or (2) through HOCl-mediated NOS heme destruction. Previously, it has been shown that NO can serve as a physiological substrate of MPO compounds I and II, and other members of mammalian peroxidase superfamily, both in the presence and absence of plasma levels of Cl^-^ (100 mM) or with a superoxide generating system, forming nitrosonium cation (NO^+^) 65. The so generated NO^+^ is highly reactive (half-life < 0.3 ns) and rapidly decays, forming NO_2_^-^ as a final product 65. These interactions also suggest a complex and interdependent relationship between NO levels and modulation of peroxidase steady-state catalysis in vivo. For instance, tracheal ring investigations have studied the potential functional consequences of peroxidase-NO interactions in asthma. NO-dependent bronchodilation of preconstructed tracheal rings was reversibly inhibited when exposed to pathological levels of peroxidase and H_2_O_2_ 89. It is possible that the interaction of NO and mammalian peroxidase serves as a regulatory pathway for the catalytic activities of those species and therefore inflammatory events. Furthermore, it has been observed that progression of COVID-19 is accompanied by impaired guanylate cyclase activetion and vascular response to endothelium-derived relaxing factor or NO 90. It is therefore tempting to speculate that peroxidases like MPO might play a role in altering guanylate cyclase activation or other NO-dependent signaling events during development of vascular disease. For instance, there has been some evidence that deficiency of NO intensifies arterial thrombosis, while NO supplementation decreases it 91.

HOCl overproduction can also mediate NOS heme destruction through a mechanism similar to its destruction of Hb heme, although this pathway's role in modulating NO bioavailability in vivo remains to be determined. NO may also react with hemoprotein model compounds, generating the corresponding Fe-NO complexes 92. NO is also a potent scavenger of a variety of radical species, such as lipid peroxyl and alkoxyl radicals 53. Thus, it follows that these systems' contribution to increased net NO consumption likely limits NO bioavailability in COVID-19 patients, subsequently causing characteristic symptoms of vasoconstriction and hypertension. Therefore, NO supplementations could be helpful in combating COVID-19 symptoms and progression, especially before severe changes have occurred in the vasculature.

## COVID-19 affects virtually every organ system

**Figure 3:**
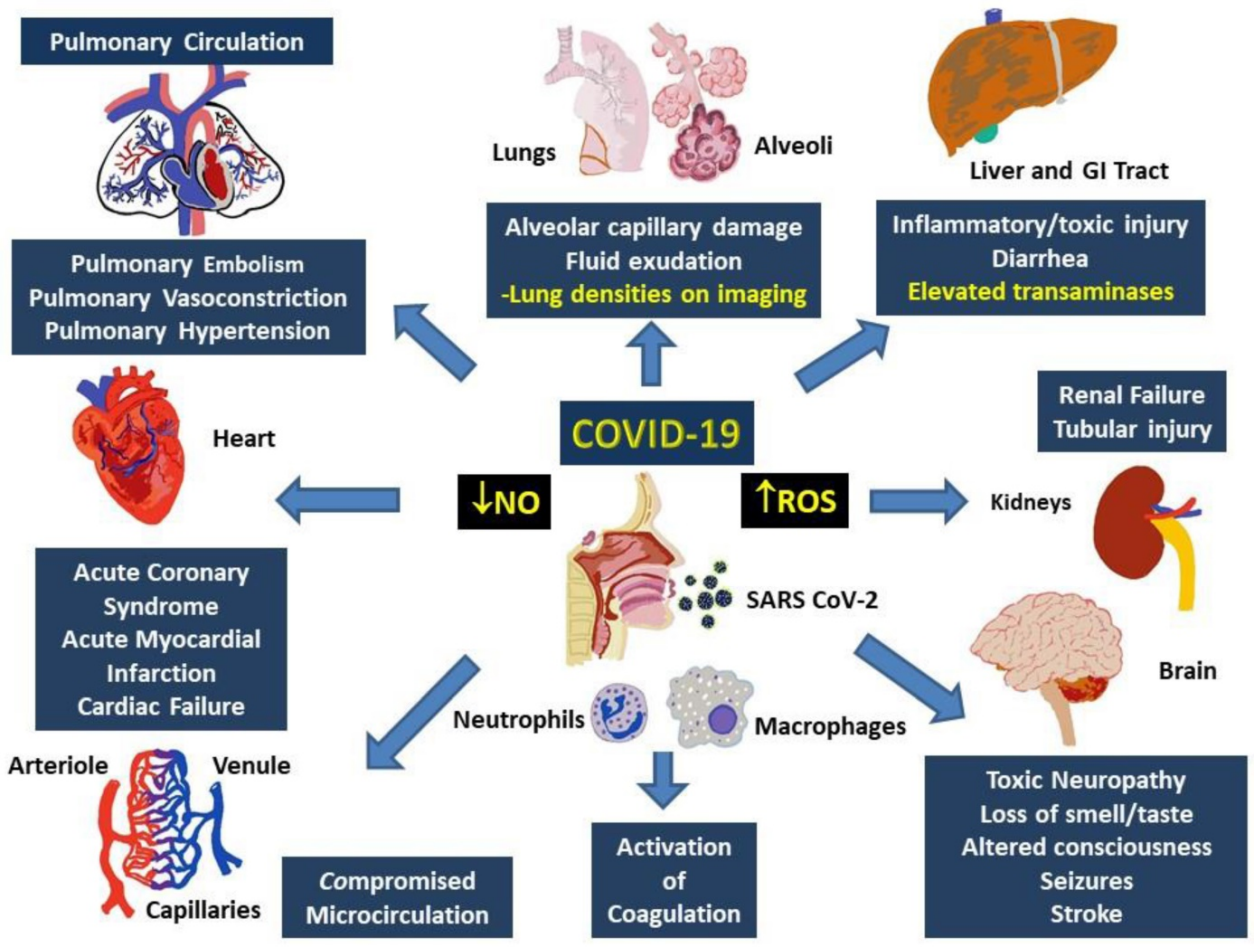
** Involvement of multiple systems in COVID-19.** Activation of the immune system following the entry into the respiratory system involves activation of neutrophils and monocytes with release of the MPO and ROS, which cause damage to both, local (alveoli and lung parenchyma) as well as distant tissues including gastrointestinal tract and liver, kidneys, brain, peripheral circulation and the heart. Damage to these tissues manifest as organ dysfunction and multi-system failure. Release of heme degradation products and iron along with ROS are particularly damaging to the kidneys, liver, heart, and brain, while inappropriate activation of the coagulation system and insufficiency of NO can result in pulmonary and peripheral vasoconstriction. Simultaneous occurrence of these events can result in severe, persistent, and potentially fatal hypoxemia.

Multisystem involvement is a hallmark of COVID-19. Although direct viral spread to distant organs is a possibility, release of inflammatory mediators and downstream messengers are also likely important effectors of physiological systems. Major physiological systems affected in COVID-19 include, primarily, the respiratory and cardiovascular system, as well as the renal, hepatic, and nervous systems (Figure 3).

In the respiratory system, histopathology indicates diffuse alveolar damage with denuded alveolar cells, interstitial mononuclear infiltrates and type II pneumocyte hyperplasia, edema, intra-alveolar proteinaceous exudate, patchy inflammation and multinucleated giant cells as well as interstitial fibrosis and intra-alveolar loose fibrous plugs 93. While some of these features represent ARDS, presence of respiratory distress with severe hypoxia and well-preserved lung mechanics indicate involvement of the pulmonary vasculature. Furthermore, absence of classical ARDS symptoms such as hyaline membrane or type II pneumocyte hyperplasia do not fully support a presentation of ARDS 94. Varying degrees of elevations in D-dimer, with normal or slightly abnormal INR, PTT, and platelet counts, as well as activation of the complementary cascades combine with elevated ROS and decreased NO paves a path for explanation of the involvement of other organ systems in COVID 19.

Cardiac involvement is noted in 20-30% of hospitalized COVID-19 patients, contributing to ~40% mortality, and prognosticates the general outlook of pathological progression 95-97. It is likely secondary to pulmonary damage, its accompanying hypoxia, and the systemic inflammatory response, as noted by elevation in the markers of inflammation including IL-6, IL-2, IL-7, TNF-α, interferon-γ inducible protein (IP)-10, monocyte chemoattractant protein (MCP)-1, macrophage inflammatory protein (MIP) 1-α, granulocyte-colony stimulating factor (G-CSF), C-reactive protein (CRP), procalcitonin, and ferritin 40, 98-100. Commonly occurring cardiovascular abnormalities in COVID-19 include hypertension, arrhythmias, acute coronary syndrome and acute myocardial infarction 101. Typical markers of cardiac injury, such as troponin I and brain natriuretic peptide (BNP), accompany these pathologies and indicate particularly increased risk of mortality 101. Other cardiovascular dysfunctions include inflammatory changes, formation of microthrombi, and/or alteration of blood viscosity, which have also been reported in patients with other acute respiratory disease, such as influenza, acute pneumonia, and H1N1, and lead to severe organ damage and eventual mortality 102, 103. In particular, altered coagulation parameters, characterized by increased d-dimer, fibrin degradation products, PTT and thrombocytopenia, predict poor prognosis and are related to elevated risk of stroke and pulmonary embolism 104.

Abnormalities of renal function are known to occur in about 75% of hospitalized patients with COVID-19, and the degree of deficiency often predicts overall disease outcome 105. COVID-19 associated renal injuries include diffuse proximal tubule injury, non-isometric vacuolar degeneration, and frank necrosis as well as hemosiderin pigmentation and pigmented casts, which are associated with heme degradation and iron toxicity 106-108. Erythrocyte aggregates obstructing capillary lumens in the absence of vasculitis, platelet aggregates, or fibrinoid aggregates were also observed 106.

Hepatic injury in COVID-19 has been reported in form of elevated liver enzymes, especially in severe cases, and in diarrheas with detection of virus in the blood 109. Other likely mechanisms could be related to hypoxia and systemic immune mediated inflammation, particularly in association with ROS elevation and/or iron toxicity 108, 110.

Nervous system damage in COVID-19 is likely similar to that found in its coronavirus cousins. In SARS-CoV infection, previous reports have indicated polyneuropathy, encephalitis, acute ischemic stroke, and demyelination 111, 112. Similarly, MERS-CoV has been linked to mental disturbances, seizures, altered consciousness, ischemic stroke, or Guillain-Barre syndrome 113, 114. About 35% of hospitalized patients with COVID-19 display neurological symptoms, including headache, altered consciousness, seizures, and sudden loss of taste and/or smell 115, 116. Autopsies have revealed cerebral edema, degeneration, and encephalitis with viral RNA present in the CSF 117, 118. Possible causative factors for neurological damage include direct infection after passage through the blood-brain barrier, immune-related hypoxic injury, infectious toxic encephalopathy, acute cerebrovascular disease, and iron toxicity resulting from heme degradation 119.

## Summary

One of the characteristic changes in the blood parameters among the patients with COVID-19 includes leukocytosis with relative neutrophilia. Activation of neutrophils can trigger various cellular mechanisms, including the release of prostanoids, lysosomal enzymes, as well as highly reactive oxygen radicals and their intermediates. Myeloperoxidases released from the azurophil granules of neutrophils play a particularly critical role by participating in the synthesis of HOCl through a reaction involving H_2_O_2_ and chloride (Cl^-^). HOCl is highly reactive and not only causes lipid peroxidation of membranes, affecting their permeability; but also contributes to oxidative modification of free functional groups, inducing changes in the functionality of proteins. HOCl degrades heme with release of Fe^2+^, which in turn participates in generation of additional ROS. Furthermore, involvement of MPO and its related mechanisms result in a decrease in nitric oxide (NO), consequently leading to vasoconstriction. Taken together, these phenomena snugly fit into the clinical pathophysiology of severe/critical COVID-19 illness, which consist of alveolar capillary damage (secondary to the production of superoxide, H_2_O_2_ and HOCl), pulmonary vasoconstriction and pulmonary hypertension (secondary to NO depletion), elevated ferritin (following release of free iron secondary to heme-degradation), and deterioration of oxygen carrying capacity (secondary to heme degradation). Production of Fe^3+^ results in formation of methemoglobin, which further decreases the affinity of Hb to oxygen, shifting the O_2_ dissociation curve further to the right and deteriorating blood O_2_ saturation while preserving the tissue levels of O_2_. This causes possible delay in appearance of the classical symptoms of hypoxia and may not reflect the severity of depletion of O_2_ saturation. Another cardiovascular feature affected by these changes is thrombosis, which is exacerbated by both accumulation of free iron and depletion of NO. Thus, a multi-hit model of MPO and its multifaceted reactions that lead to production of ROS such as HOCl, O_2_•^-^, H_2_O_2_ and •OH, which then mediate decrease O_2_ diffusion, carriage, and delivery through interaction with heme proteins and other players could explain the rapid evolution of respiratory failure and subsequent mortality in patients in critical stages of COVID-19. Understanding these mechanisms can provide clues to therapies to prevent severe morbidity and mortality among patients affected with COVID-19.

## Abbreviations

COVID-19: Coronavirus disease-19
ROS: Reactive oxygen species
MPO: myeloperoxidase
H_2_O_2_: hydrogen peroxide
HOCl: hypochlorous acid
O_2_•^-^: superoxide
•OH: hydroxyl radical
